# Eliminating the effect of pathomorphologically formed sperm on resulting gravidity using the intracytoplasmic sperm injection method

**DOI:** 10.3892/etm.2014.1522

**Published:** 2014-02-06

**Authors:** EVA BLAHOVÁ, JAN MÁCHAL, LADISLAV MÁCHAL, IRENA MILAKOVIĆ, ŠÁRKA HANULÁKOVÁ

**Affiliations:** 1 Department of Animal Reproduction, Mendel University in Brno, Brno 61300, Czech Republic; 2 Department of Pathological Physiology, Faculty of Medicine, Masaryk University, Brno 62500, Czech Republic

**Keywords:** intracytoplasmic sperm injections, sperm morphology, pregnancy rate, reproduction rate

## Abstract

The aim of the present study was to test whether it is possible to eliminate a high percentage of morphologically abnormal sperm in male ejaculate by assisted reproduction using the intracytoplasmic sperm injection (ICSI) method. Treatment success was evaluated by comparing fertilization, clinical pregnancy and reproduction rates between males with heavy teratospermia (≤1% morphologically normal spermatozoa) and males with a higher percentage (>1%) of normal sperm. In total, 174 patients who had previously undergone 174 ICSI cycles (1 per each pair) were evaluated retrospectively. In the group of patients with heavily impaired sperm morphology (n=37), the percentage of normal spermatozoa was ≤1%. In the second group, males with >1% normal spermatozoa (n=137) were considered as patients with mildly impaired sperm morphology. The results of partner fertilization in these two groups were compared and a lower number of fertilized oocytes was identified in the patients with heavily impaired sperm morphology (P=0.038). However, neither the gravidity nor the take-home baby rates of the partners differed between the patients with mildly and heavily impaired sperm morphology. Trends opposite to that for fertilization were observed for gravidity and delivery [odds ratio (OR), 0.62; 95% confidence interval (CI), 0.29–1.30; OR, 0.55; 95% CI, 0.26–1.24, respectively]. This indicates that the lower number of fertilized oocytes was not associated with the overall outcome of fertilization and that patients with heavily impaired sperm morphology experience the same benefit from ICSI as patients with mildly impaired sperm morphology.

## Introduction

Morphological abnormalities of spermatozoa are often identified in males with problematic fertility. Male infertility may be classified as oligoasthenoteratozoospermia or azoospermia. The quality of spermatozoa has an essential effect on the fertilization of the oocyte and on the subsequent evolution of the embryo. A direct correlation exists between abnormal sperm and embryo morphology at the later stage of cleavage (1). The first two cycles of embryo cell cleavage are controlled by maternal factors, whilst the paternal effect begins to apply in the embryo from the four-cell stage (2). The quality of DNA in sperm is evaluated as an absence/incidence of fragmentations in late embryonic development, which is the late paternal effect (3–5).

The first pregnancy and birth of a child following the application of the intracytoplasmic sperm injection (ICSI) method was recorded in 1992 (6). ICSI has increased the success rates of *in vitro* fertilization (IVF) treatment in couples with the male sterility factor. The fertilization rate following ICSI has been reported to be significantly higher compared with that of the subzonal insemination method (7). ICSI has been found to result in higher fertilization and pregnancy rates; it has been shown to be successful in couples with unexplained infertility (8), boundary spermiogram values (9) and immunological infertility (10), and couples who have experienced repeated failures following conventional treatment by IVF (11). Common indications for use of the ICSI method are low parameters of spermatozoa in the ejaculate. The ICSI method with ejaculated sperm may be successfully used in patients with a low count of morphologically high-quality and motile sperm in the ejaculate. Riedel *et al* (12) established minimum andrological parameters for *in vitro* fertility in conventional IVF, specifying a 5×10^6^ cm^−3^ total count, 30% progressive motility and 30% normal morphology; males exhibiting worse parameters in the ejaculate had an very low prognosis of successful medical treatment. The ICSI method represents an effective procedure for this type of male infertility.

In the present study, fertilization, pregnancy and take-home baby rates were analyzed following ICSI in males with a high percentage of morphologically abnormal spermatozoa. Teratospermia is one of the key parameters in the selection of sperm suitable for ICSI. The aim of the present study was to ascertain whether ICSI is a suitable treatment perspective for infertile males with heavy teratospermia (≤1% normal spermatozoa).

## Materials and methods

### Patients

ICSI was performed between January 2008 and February 2009 to assist reproduction in 174 infertile couples at the Assisted reproduction clinic (Brno, Czech Republic). Success of the treatment was evaluated from the male viewpoint, as the study focused on male infertility. In total, 174 males were subjected to ICSI cycles. The male patients were divided into a group of 137 individuals with mildly impaired or normal sperm morphology (>1% of normal spermatozoa) and a group of 37 individuals with heavily impaired sperm morphology (≤1% of normal spermatozoa). The mean age of the male patients was 34.9±2.8 years and the age of their female partners was 30.5±2.7 years. Informed consent was obtained from all participants. The study was approved by the Ethics Committee of the Faculty of Medicine (Masaryk University, Brno, Czech Republic).

### Semen analysis

Spermatozoa were obtained through masturbation following sexual abstinence for 3–5 days. Prior to analysis, samples were incubated for 20 min at 37°C for fluidization. The concentration, motility and morphology of the sperm were assessed according to the guidelines of the World Health Organization (4th edition, 1999). Samples were visually assessed under a microscope.

### Sperm processing

Spermatozoa gained from ejaculation were processed using the swim-up method (13) and incubated at a temperature of 37°C with 5% O_2_, 6% CO_2_ and 89% N_2_ in Sydney IVF Sperm Medium (Cook Medical, Brisbane, Australia).

### Ovarian stimulation and oocyte retrieval

Ovarian stimulation was induced by a long protocol in all 174 cycles using triptorelin [a gonadotropin-releasing hormone agonist (GnRHa); Ferring Pharmaceuticals, Saint-Prex, Switzerland], Metrodin [follicle-stimulating hormone (FSH); Serono, Geneva, Switzerland] and Humegon [human menopausal gonadotropin (hMG); Organon Laboratories Ltd., Hoddesdon, UK). Subcutaneous triptorelin application was initiated from the mid-luteal phase of the previous cycle and continued for 14 days until sufficient downregulation of the pituitary was achieved. The development of follicles was stimulated by FSH and hMG injections. The dose of gonadotropins was individualized, and selected according to the age of the female patient, previous stimulation and response to stimulation. Ovulation was induced by injecting 5,000 IU human chorionic gonadotropin (hCG) in the form of Pregnyl (Organon Laboratories Ltd.) when the two largest follicles were >18 mm. Oocytes were sampled 36 h following hCG application under general anesthesia using ultrasound control.

### Oocyte handling

Cumular cells were removed from the oocytes with a denudation pipette (1–2 h following oocyte sampling by ovum pick up) using 80 IU/ml hyaluronidase (in Sydney IVF Fertilization medium) for 10–15 sec. Following the partial removal of cumular cells, the oocytes were further denudated (Sydney IVF Fertilization medium) until complete denudation. The denudated oocytes were placed in a cultivation box (Heracell; Thermo Fisher Scientific, Waltham, MA, USA) under conditions of 37°C, 5% O_2_, 6% CO_2_ and 89% N_2_, until required for ICSI. The sperm were injected into the oocytes 2–3 h following ovum pick up. Oocytes used for ICSI were in metaphase II (MII) after extrusion of the first polar body.

### ICSI procedure

ICSI was conducted using an inverted microscope (Olympus, Tokyo, Japan) with a micro-manipulator (Research Instruments, Falmouth, UK) and injectors (Eppendorf, Hamburg, Germany). During ICSI, oocytes were maintained in Sydney IVF Fertilization medium, and Sydney IVF PVP (Cook Medical) was used for spermatozoa. The oocytes were placed individually into 10-μl micro-drops of Sydney IVF Fertilization medium and one micro-drop with the Sydney IVF PVP medium was injected with a 2-μl suspension of spermatozoa. Sperm were selected, immobilized, sucked into the ICSI pipette and inserted into the oocyte cytoplasm under a microscope at ×400 magnification, using Hoffman modulation contrast. Prior to injection, the morphological structure of the head, neck and tail of the sperm was assessed, as well as the possible occurrence of vacuoles in the sperm head. Following ICSI, the oocytes were transferred into Sydney IVF Cleavage medium (Cook Medical) and deposited in the cultivation box.

### Assessment of fertilization, embryo cleavage and establishment of pregnancy

After 16–18 h, oocytes were checked to verify fertilization. Fertilized oocytes were separated and tested for the occurrence of the two-pronuclei (2PN) stage. The cleavage phase of the embryo was established 25–27 h following oocyte fertilization and early embryo cleavage was assessed (14). The early paternal effect of the sperm is demonstrated prior to the main activation of embryonic genome expression, as it starts between the fourth and the eighth cell stage of embryo preimplantation development (3,4). Embryos of the highest quality were transferred within 72–96 h from the sampling of oocytes using a Wallace catheter (1816N; H.G. Wallace Ltd., London, UK). Parameters evaluated in the embryos included the number and regularity of blastomeres, as well as the incidence or absence of fragments and vacuoles (15). The mean number of transferred embryos was two per transfer. The luteal phase was supported by progesterone in the form of Utrogestan (Besins Manufacturing Belgium S.A., Drogenbos, Belgium) 2× 200 mg daily or by injecting Agolutin (Biotika a.s., Slovenská L̓upča, Slovakia) 50 mg daily and 1,500 IU Pregnyl (N.V.Organon, Oss, The Netherlands on days 1, 4, 7 and 9 following embryo-transfer. In the case of positive chemical gravidity, HCG was detected on day 14 following embryo-transfer. Clinical gravidity was defined as an intrauterine identification of the gestational sac with a heart function. Abortion was defined as gravidity terminated prior to week 20 of pregnancy.

### Statistical analysis

Comparisons were made between pregnancy and reproduction rates. The normality of data was tested by the Anderson-Darling normality test and by visual inspection of histograms. As specific parameters exhibited a non-normal distribution, the Mann-Whitney U-test was used to compare continual data. Fisher’s exact test was applied to compare categorical data. P<0.05 was considered to indicate a statistically significant difference.

## Results

Oocytes injected in the MII phase totaled 2,811 and subsequent fertilization (2PN) was recorded in 2,303 oocytes (82%). Transfer was implemented in all 174 couples and the total number of transferred embryos amounted to 349 with an average count of two embryos per transfer. Clinical gravidity per transfer was achieved in 92 cases (53%). The resulting number of births was 83 (48%) with 108 live-born infants.

Table I summarizes the characteristics of the patients. Table II summarizes characteristics and outcomes of the patients and is divided into groups of males with mildly and heavily impaired sperm morphology. Statistical evaluations were performed of the age of male patients, mean count of oocytes sampled from their female partners, number of oocytes in the MII phase suitable for fertilization, number of fertilized oocytes, number of clinical gravidities and number of deliveries of live-born children. Table II indicates that while the number of fertilized oocytes in the patients with heavily impaired sperm morphology was significantly lower (P=0.038), neither gravidity nor delivery of the partners differed compared with those in the patients with mildly impaired or normal spermatozoa. Trends opposite to that for fertilization were recorded for gravidity [odds ratio (OR), 0.62; 95% confidence interval (CI), 0.29–1.30] and delivery (OR, 0.55; 95% CI, 0.26–1.24). These results indicate that the lower number of fertilized oocytes was not associated with the overall result.

**Figure 1:**
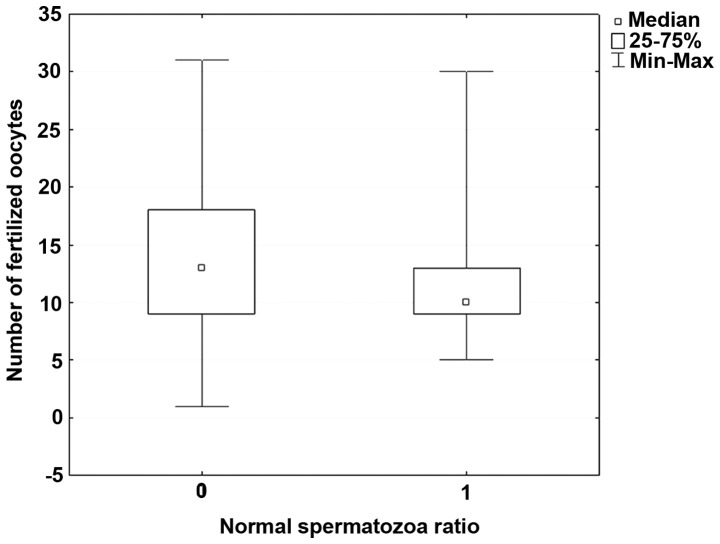
Number of fertilized oocytes in the partners of patients with heavily and mildly defective spermatozoa.

The present study indicates that there was a significant difference in fertilization rates between the group of males with heavily impaired sperm morphology and those with mildly impaired sperm morphology. The result of a successful treatment was pregnancy and the birth of a healthy child. Statistical evaluation showed no significant difference between the two groups in this respect (Fig. 1).

## Discussion

The prospects of producing biological offspring, in couples who previously had no likelihood of doing so, have considerably increased since the ICSI method was introduced in 1992 (first gravidity and delivery of a child following ICSI applied in a female). Following the first successful pregnancy and birth of a healthy child using the ICSI method, the procedure began to be widely used to assist fertilization, particularly in couples affected by male infertility (6). Gradual improvement of procedures in the evaluation of spermatozoa and micromanipulation techniques has opened new horizons for the successful assistance of reproduction in cases of male infertility. Compared with the conventional method of IVF, ICSI yields higher fertilization rates, as well as higher counts of cleaved embryos (16). One of the main differences between conventional IVF and the ICSI method is the ability to select just one sperm and insert it mechanically into the egg cytoplasm (17). Studies by Aytoz *et al* and Palermo *et al* found no significant difference between the pregnancy rates for normal and abnormal ejaculates using the ICSI method (18,19), consistent with the results of the present study. The method of selecting a suitable sperm markedly increased the count of fertilized oocytes in IVF/ICSI treatment and provided a general solution for the problem of heavy teratospermia in males who had no likelihood of achieving a successful treatment result. Therefore, the requirements for sperm donors have been markedly reduced.

The correlation between morphology and the low success of conventional IVF treatment has been clearly ascertained (20,21). The normal morphology of sperm is important for binding and penetration through the zona pellucida. Injection of one sperm through the zona pellucida and oolemma into the egg cytoplasm facilitates penetration of the egg, even for sperm previously incapable of overcoming these barriers during normal IVF fertilization. A range of studies have indicated that when using the ICSI method, the high percentage of morphologically abnormal sperm in the ejaculate has no essential effect on the outcome of fertilization, transfer of high-quality embryos and gravidity (10,22–25). A study by De Vos showed a lower fertilization rate of oocytes for patients with abnormal spermatozoa but a comparable quality of embryos. Lower implantation and clinical gravidity rates for abnormal sperm were also observed (26). The present study using ICSI observed that lower fertilization rates were obtained in males with heavy teratospermia; however, this did not affect subsequent implantation, clinical gravidity and delivery of a healthy child. Fishel *et al* (27) conducted a study to compare IVF and ICSI methods using partner sperm and at the same time using donor sperm for IVF in couples with unexplained infertility. The highest fertilization incidence was observed in ICSI with the partner sperm. The conclusion of the study was that ICSI is an effective method of achieving fertilization in higher counts of oocytes, maximizing the number of embryos and minimizing the risk of fertilization failure in the treatment of infertility.

The results of the present study indicate that fertilization of oocytes is less effective in patients with poor morphological quality of sperm cells, compared with the effectiveness in patients with mildly impaired morphology. However, pregnancy and reproduction rates were independent of sperm morphology when ICSI was used. These results are in concordance with previously published studies. Microscopic selection of a sperm with ‘normal’ morphology during the ICSI procedure achieves excellent results, even in heavy teratospermias (28). Following introduction of the ICSI method, 2,000 children conceived using this method have been born; these individuals have not exhibited a higher incidence of malformations compared with those in children born following conventional IVF or in the normal population (29). However, this conclusion does not correspond with later results published by Hansen *et al* and Bonduelle *et al* (30,31) or with a subsequent study by Davies *et al* (32). Hansen *et al* observed that the incidence of inborn developmental defects diagnosed in the first year of life in children conceived with ICSI was double that in children conceived naturally (30). Factors that may increase the risk include the relatively high age of infertile couples, causes of infertility and the effect of drugs used to induce ovulation or to maintain gravidity in the initial stages of embryonic development, as well as factors connected with methods of assisted reproduction, freezing and unfreezing of embryos, late fertilization of oocytes and polyspermy. In the aforementioned study, a higher incidence of cardiovascular, urogenital, chromosomal and musculoskeletal defects was recorded when the ICSI method was used in assisted reproduction. Following the adjustment for parental risk factors, the incidence of developmental defects was not significantly higher in IVF, while the risk of ICSI-associated defects remained significantly higher following adjustment. An analysis of perinatal results from singleton pregnancies identified significant differences in the gestation age, birth weight, body length, head circumference and Apgar score in children conceived using ICSI when compared those in children born following spontaneous pregnancies (33). The international study by Bonduelle *et al* focused on children born following ICSI and their subsequent 5-year development (31). During these five years, these children showed a greater probability of requiring physiotherapy and speech therapy and showed a higher susceptibility to respiratory, dermatological or gastrointestinal infections, resulting in a higher need for various types of surgery. The extensive observational study by Davies *et al* (32) corroborated the conclusions of the two aforementioned studies (30,31) with regard to the increased risk of defects in newly born children conceived through assisted reproduction compared with the risk in children from spontaneous conception. The study by Davies *et al* is more comprehensive and includes 6,163 children conceived through assisted reproduction and evaluated until the age of five years. The increased risk of inborn IVF-associated defects is not significant following adjustment for parental risk factors, while in the ICSI method the higher risk persists. Notably, results from a comparison between fresh and frozen embryos used in IVF and ICSI cycles indicate that embryos transferred following thawing in the two methods did not show a higher incidence of developmental defects following birth than infants from natural conception. These results may be explained by the theory that embryos are unlikely to survive the demanding process of freezing and unfreezing unless they are of high quality (34). The process of mechanical injection of a selected sperm into the egg cytoplasm in ICSI avoids all natural barriers of spontaneous fertilization. As shown in the study by Fishel *et al* (27), in conventional IVF using high concentrations of donor sperm, when there is no intervention in the process of sperm penetration into the egg, the fertilization rate was significantly lower than that in the ICSI method with partner sperm, despite the partner sperm not exhibiting the high quality of the donor sperm.

The present study ended at the delivery of a full-term infant. Developmental defects of the fetuses were not observed, which may have been due to the low age of the females included in the study (30.5±2.7 years). Data concerning the subsequent development of the children are not available. Although the ICSI method is often the only option for gravidity and delivery of biological offspring, certain studies indicate that ISCI almost doubles the risk of inborn developmental defects in children (30,32). For males with oligoasthenoteratospermia, ICSI treatment is often the only potentially successful method of producing biological offspring. Despite infertile parents having a number of age-associated negative factors that may increase the risk of developmental defects in the fetus, medication and ICSI remain the most efficient method for males with heavy teratospermia to achieve pregnancy and the birth of a child, as shown in the present study. The development of a new technique of continual monitoring in the cultivation of embryos may provide an improved method for the selection of the embryos that are most suitable for transfer, which may subsequently decrease the incidence of fetal developmental defects (35).

## Figures and Tables

**Table I:** Basic characteristics of the male subjects and their partners during intervention.

Characteristics	Value
Male age, years	34.9±2.8
Female age, years	30.5±2.7
Normal spermatozoa, %	11 (3–21)^a^
Oocytes, n	25±8
Mature oocytes, n	16±6
Fertilized oocytes, n	12 (9–17)^a^

Normal distribution values are listed as mean ± SD.

a Non-normal distribution values are listed as median (lower quartile-upper quartile).

**Table II:** Characteristics and the outcome of fertilization in patients with mildly and heavily defective spermatozoa.

Morphology	Heavily defective (≤1% normal spermatozoa)	Mildly defective (>1% normal spermatozoa)	P-value
Age, years	35.3±3.1	34.8±2.7	0.339
Oocytes, n	26±7	24±8	0.271
Mature oocytes, n	15±6	16±6	0.192
Fertilized oocytes, n	10 (9–13)^a^	13 (9–17)^a^	0.038
Pregnancy rates, present/absent	23/14	69/68	0.266
Delivery, present/absent	22/15	61/76	0.138

Values with normal distribution are listed as mean ± SD.

a Non-normal distribution values are listed as median (lower quartile-upper quartile).
